# Abundant Genetic Diversity and Extensive Differentiation among Geographic Populations of the Citrus Pathogen *Diaporthe citri* in Southern China

**DOI:** 10.3390/jof7090749

**Published:** 2021-09-13

**Authors:** Tao Xiong, Yating Zeng, Wen Wang, Pudong Li, Yunpeng Gai, Chen Jiao, Zengrong Zhu, Jianping Xu, Hongye Li

**Affiliations:** 1 Ministry of Agricultural and Rural Affairs Key Laboratory of Molecular Biology of Crop Pathogens and Insect Pests, Institute of Biotechnology, Zhejiang University, Hangzhou 310058, China; 21816084@zju.edu.cn (T.X.); 21816172@zju.edu.cn (Y.Z.); 11916090@zju.edu.cn (W.W.); pdli@zju.edu.cn (P.L.); gaiy@zju.edu.cn (Y.G.); biochenjiao@zju.edu.cn (C.J.); zrzhu@zju.edu.cn (Z.Z.); 2 Hainan Institute, Zhejiang University, Sanya 572000, China; 3 Department of Biology, McMaster University, Hamilton, ON L8S 4K1, Canada

**Keywords:** citrus melanose, simple sequence repeats, geographic differentiation, genetic clusters, mating type, linkage disequilibrium, sexual reproduction

## Abstract

The fungal pathogen *Diaporthe citri* is a major cause of diseases in citrus. One common disease is melanose, responsible for large economic losses to the citrus fruit industry. However, very little is known about the epidemiology and genetic structure of *D. citri*. In this study, we analyzed 339 isolates from leaves and fruits with melanose symptoms from five provinces in southern China at 14 polymorphic simple sequence repeat (SSR) loci and the mating type idiomorphs. The genetic variations were analyzed at three levels with separate samples: among provinces, among orchards within one county, and among trees within one orchard. The five provincial populations from Fujian, Zhejiang, Jiangxi, Hunan, and Guizhou were significantly differentiated, while limited differences were found among orchards from the same county or among trees from the same orchard. STRUCTURE analysis detected two genetic clusters in the total sample, with different provincial subpopulations showing different frequencies of isolates in these two clusters. Mantel analysis showed significant positive correlation between genetic and geographic distances, consistent with geographic separation as a significant barrier to gene flow in *D. citri* in China. High levels of genetic diversity were found within individual subpopulations at all three spatial scales of analyses. Interestingly, most subpopulations at all three spatial scales had the two mating types in similar frequencies and with alleles at the 14 SSR loci not significantly different from linkage equilibrium. Indeed, strains with different mating types and different multilocus genotypes were frequently isolated from the same leaves and fruits. The results indicate that sexual reproduction plays an important role in natural populations of *D. citri* in southern China and that its ascospores likely represent an important contributor to citrus disease.

## 1. Introduction

*Diaporthe citri* (anamorph *Phomopis citri*) is one of the most important pathogenic fungi on citrus. It induces melanose on the leaves, twigs, and fruits; stem-end rot of postharvest fruits; and gummosis and blight of shoots, perennial branches, and trunks of citrus. Among these diseases, melanose brings perhaps the most significant economic loss since it significantly reduces the marketability of citrus fruits [1,2,3,4,5]. This fungal pathogen is widespread in humid citrus-producing areas, except for California in the US and Europe [5,6]. All citrus species are susceptible to *D. citri* infection, with grapefruit and lemons being the most severely affected [5].

*D. citri* produces both sexual spores (ascospores) and asexual spores (conidia) on dead twigs and branches, but it is not known to sporulate on living tissues [7,8]. Several studies have reported perithecia, the sexual reproductive organ of *D. citri,* on decaying wood on the ground or on the dead branches on trees [7,8,9]. Once mature, ascospores could eject from perithecia, become airborne, and disseminate to new niches. However, perithecia are not commonly found in nature, suggesting ascospores may play a relatively minor role in the epidemiology of melanose. Instead, the asexual reproductive organs pycnidia are often observed abundantly on dead twigs, from which the conidia are often shown as slimy masses secreted from these twigs during the citrus growing season, especially after rainfall [5,7,8]. Conidia can spread by rain-splash to leaves, twigs, and fruits, causing short-distance dispersals of this pathogen to other sites, either within the same tree canopy or to adjacent trees. Thus, the reproductive cycle of this fungus has been primarily considered as occurring on dead twigs and decaying woods, and with the living infected tissues playing a minor role in its reproduction in nature [3,5,7,8].

The genus *Diaporthe* belongs to the order Diaporthales in class Sordariomycetes of the Ascomycota phylum. Species in *Diaporthe* usually have broad host ranges, with most species capable of infecting multiple plants [10,11,12]. Aside from *D. citri*, other species in *Diaporthe* can also cause diseases such as necrosis spots, diebacks, cankers, decay, or wilts on different plant hosts including cultivated crops, trees, and ornamental plants, which could lead to host tree deaths or significant economic losses. Examples of diseases caused by *Diaporthe* species on common crops include the soybean stem blight by *Diaporthe phaseolorum*; stem canker of sunflower by *Diaporthe helianthi*; leaf spot, swelling arm, and perennial cankers of grapevines by multiple *Diaporthe* species; and branch cankers and fruit stem-end rot of avocado by *Diaporthe foeniculina* and *Diaporthe sterilis* [6,11,12,13,14,15]. Although the importance of *D. citri* and other pathogens in the *Diaporthe* genus is well-recognized, our knowledge about their diversity, population genetics, reproduction strategies, and pathogenicity is very limited [16,17]. Knowledge on their population biology and mode of reproduction in nature can help us understand many issues, including the mechanisms of pathogenesis and host range, the development of fungicide resistance, and the effectiveness of phytosanitary measures. Such knowledge can facilitate the development of sustainable management strategies against this and other groups of plant fungal pathogens [18,19,20].

Recently, with the analyses and release of the genomic sequences of 12 species in the genus *Diaporthe*, including those of three isolates of *D. citri* (ZJUD2, ZJUD14, and Q7), the heterothallic mating system and the structures and DNA sequences of both mating types (MAT) were elucidated [21,22]. DNA sequence analyses revealed that each strain of *D. citri* contained only one mating type (MAT) idiomorph: strain ZJUD14 contained *MAT1-1* and strains ZJUD2 and Q7 contained *MAT1-2* [22]. These genome sequences allow us to develop genetic markers to explore the population structure, genetic diversity, and mating type distributions in natural populations of *D. citri*. Specifically, in this study, we aimed to analyze the patterns of genetic variation at three spatial scales: (i) at the provincial level among five provinces (Fujian, Zhejiang, Jiangxi, Hunan, and Guizhou) in southern China, (ii) among four orchards within one county in Hunan Province, and (iii) among five trees from a single orchard, also in Hunan. In total, we obtained 339 isolates and analyzed their genotypes at 14 newly developed polymorphic simple sequence repeat (SSR, microsatellites) markers. In addition, the mating type of each isolate was determined using a mating type-specific PCR. We hypothesize that geographic separation plays a major role in genetic relationships among subpopulations of *D. citri*. Specifically, we hypothesize that the five provincial populations of *D. citri* in southern China will be genetically differentiated, while relatively limited differences will be found among subpopulations of *D. citri* from different orchards within the same county and among samples from different trees within the same orchard. In addition, based on what has been reported before about the dominance of the asexual reproduction of *D. citri* in nature [5,8,9], we hypothesize that there will be abundant evidence for asexual reproduction, including evidence for linkage disequilibrium, with multiple isolates sharing the same genotypes, especially among *D. citri* isolates from the same tree and the same orchard. Furthermore, based on the observed broad host range of *D. citri* [10,11,12], we hypothesize that there will be limited host tree-based and host organ-based population structure of this fungal pathogen. To test the third hypothesis, melanose-diseased samples from two host tree species from within the same county were analyzed. Similarly, melanose-diseased leaves and fruits from the same trees within a single orchard were sampled and analyzed. Together, these hypotheses were tested using a variety of samples and statistical and population genetic methods.

## 2. Materials and Methods

### 2.1. Isolate Collection

In total, 339 single-conidial isolates of *D. citri* were collected between June and September in 2019 from *Citrus reticulata* (Ponkan) and *C. sinensis* (navel orange) in 21 orchards in five provinces of southern China (Table 1). These samples were collected to address strain relationships and population genetic structures at three spatial levels, as described below.

The first level was the finest scale where we sampled extensively among five trees (the trees were at least 10 m from each other) in one orchard in Luxi county, Hunan Province. For each tree, five leaves and five fruits with melanose symptoms were sampled and 10 melanose spots per leaf/fruit were individually treated for isolation of *D. citri*. All host trees in this orchard were *C. reticulata* and a total of 95 isolates were obtained for study.

The second spatial level analyzed *D. citri* samples from four orchards within Luxi county in Hunan Province. Two of the four orchards had *C. reticulata* while the other two were planted with *C. sinensis*. Here, four leaves or fruits with melanose symptoms were randomly collected from each of 10 random trees (at least 10 m apart) in each orchard. A total of 102 isolates were obtained from these four orchards. At this level, we analyzed the potential effects of intermediate spatial separation and host tree species on the total genetic variations.

The third level is the largest spatial scale at the provincial level, where 142 isolates were collected from 18 orchards of *C. reticulata* located in five provinces. These included 24 isolates from four orchards in Fujian (FJ) Province, 30 isolates from three orchards in Guizhou (GZ) Province, 30 isolates from four orchards in Jiangxi (JX) Province, 28 isolates from five orchards in Zhejiang (ZJ) Province, and 30 isolates from two orchards in Hunan (HN) Province. Similar to the two smaller scales, each isolate was derived from an independent melanose spot on either leaves or fruits. Here, the isolates were derived from at least five trees in each orchard. For each tree, 1–4 leaves or fruits with melanose symptoms were randomly sampled for isolation of *D. citri*. However, to reduce the potential over-representation of isolates from certain trees, only one isolate from each tree was randomly chosen for genotyping and subsequent data analyses.

To minimize confounding factors, the three levels of analyses used separate samples. Strains of *D. citri* were isolated following the method described previously [2]. Cultures were stored at 4 °C. For long-term storage, the isolates were kept in 30% glycerin in a −80 °C freezer.

### 2.2. DNA Extraction

For each isolate, its genomic DNA was extracted from mycelia scraped off with a sterile blade from a colony growing on PDA at 25 °C for about 7 days. The scraped mycelia were lyophilized, and then the DNA was extracted using a SIMGEN Fungi Kit (Simgen, Hangzhou, China) according to the manufacturer’s protocol. The extracted genomic DNA samples were quantified using a Nanodrop spectrophotometer (Nanodrop Products, Wilmington, DE, USA) and stored in sterile water in a −20 °C freezer.

### 2.3. Identification of D. citri-like Isolates

The obtained *D. citri*-like isolates were confirmed based on their colony morphology, microscopic conidia features, and molecular characteristics [2]. Specifically, we used *D. citri*-specific primer pair Dc-F/Dc-R (5′-CCCTCGAGGCATCATTAC-3′/5′-ATGTTGCAGATGGTCAAATGG-3′) designed based on sequence variations in the *Tub* gene among 21 *Diaporthe* species from citrus [2]. Each PCR reaction was conducted in a final volume of 20 µL, containing 1 µL of template genomic DNA (~20 ng), 0.8 µL forward primer, 0.8 µL reverse primer, 10 µL of 2 × Taq Plus Master Mix (Vazyme Biotechnology Co. Hangzhou), and 7.4 µL sterile water. PCR cycling condition was performed as follows: 94 °C for 3 min, 31 cycles of 94 °C for 30 s, 60 °C for 30 s, 72 °C for 1 min, with a final extension of 3 min at 72 °C. The PCR products were analyzed by electrophoresis at 120 V for 30 min in a 1% (*w*/*v*) agarose gel and visualized under UV light after ethidium bromide staining. Genomic DNAs from two strains (ZJUD2 and ZJUD14), which were identified as *D. citri* [2], were used as positive controls. The genomic DNAs of *Diaporthe citriasiana* strain ZJUD31 and *D. citrichinensis* strain ZJUD40 [2] were used as negative controls. These four reference isolates are stored at the Institute of Biotechnology at Zhejiang University (Hangzhou, Zhejiang Province, China).

### 2.4. SSR Marker Development and Screening

The genome sequence of *D. citri* strain ZJUD2 [22] was screened using the online tool Batchprimer 3 (https://wheat.pw.usda.gov/demos/BatchPrimer3/, accessed on 5 November 2020) [23] to identify potential SSR markers. SSRs containing 2 to 54 tandem repeats of di- to hexa-nucleotides as well as their flanking regions were retrieved. Primers were designed using Batchprimer 3 following the default setting to obtain amplification products in the range of 100 bp to 250 bp to allow optimum genotyping and scoring [24]. The selected primers were screened for levels of polymorphism and amplification success using a subset of 12 isolates of *D. citri* (ZJUD2, ZJUD6, ZJUD14, ZJUD26, ZJUD13, ZJUD24, ZJUD22, ZJUD28, ZJUD19, ZJUD21, ZJUD15, and ZJUD8) [2], which were kept at the Institute of Biotechnology at Zhejiang University. The PCR cycling conditions used the following touch-down protocol: 94 °C for 5 min, 10 cycles of 94 °C for 30 s, 60 °C for 30 s, with a decrease in annealing temperature of 1 °C in each cycle, and 72 °C for 30 s, followed by 25 cycles of 94 °C for 30 s, 52 °C for 30 s, and 72 °C for 30 s, with a final extension of 10 min at 72 °C. The PCR products were separated on polyacrylamide gels and stained with ethidium bromide before visualization under UV light. Loci were considered polymorphic if two or more alleles were observed among the 14 isolates evaluated.

Based on amplification success and the level of polymorphisms, we selected 14 markers to genotype the subset of 14 isolates from our recent collection. The optimum annealing temperature of the selected 14 pairs of primers was derived by gradient PCR using the genome DNA of ZJUD2. The PCR reaction system was the same as described above. The PCR cycling conditions were: 94 °C for 5 min, 31 cycles of 94 °C for 30 s, 50~62 °C for 30 s, 72 °C for 30 s, with a final extension of 5 min at 72 °C. The PCR products were separated and visualized as described above for confirmation of amplification success and polymorphisms among the 14 representative strains. All 14 primer pairs successfully amplified a single product from each of the 14 representative strains. These 14 primer pairs were then used to genotype all 339 isolates following protocols described below.

### 2.5. SSR Loci Genotyping

For each of the 14 SSR markers, the forward primer was labeled with either FAM, HEX, or TAMRA fluorophore. The PCR reaction system was the same as described above. PCR cycling was performed as follows: 94 °C for 5 min, 35 cycles of 94 °C for 30 s, 53, 55, or 56 °C (depending on the primer pair) for 30 s, 72 °C for 30 s, with a final extension of 10 min at 72 °C. The amplicons were separated using capillary electrophoresis. Capillary electrophoresis was performed in multiplex reactions of three amplicons with three different fluorescent dyes per reaction. The PCR products were analyzed using an ABI 3730xl Genetic Analyzer (Applied Biosystems, Foster City, CA, USA) with a GS500LIZ Size Standard (Applied Biosystems). The output files were analyzed using GeneMapper v4.1 software (Applied Biosystems) to identify the lengths of amplified fragments at each locus.

### 2.6. Diversity Characteristics of the 14 SSR Markers

For most diversity estimates, two sets of samples were analyzed: non-clone-corrected (i.e., the raw sample) and clone-corrected. For clone-corrected datasets, only a single isolate of each multilocus genotype (MLG) was retained from each sub-population. Isolates with the same alleles at all loci were considered as belonging to the same clone. Clone-corrected datasets were used to minimize potentially distorted estimates of allelic diversity due to differences in sampling efforts and in *D. citri* isolation successes [25].

To determine gene diversities of the subpopulations and populations at different spatial scales, the following diversity estimates were calculated using the program GenAlEx v. 6.5.2 [26]: number of alleles (Na), number of private alleles, effective number of alleles, Shannon’s polymorphism information index [27], Nei’s gene diversity [28], and unbiased gene diversity [29].

To determine the genotypic diversities in subpopulations and populations at each of the three spatial scales, the number of MLGs was determined in R v. 3.6.3 (R Core Team 2013) [30] using the analysis package *poppr* [31]. Genetic and genotypic diversities were not calculated for samples from individual trees and orchards due to their small numbers of isolates. To assess the power of our SSR markers for discriminating between strains and multilocus genotypes in the dataset, a rarefaction curve was generated using *poppr* [31].

### 2.7. Genetic Structure Analysis of D. citri Populations

Population genetic structure was analyzed using several approaches. In the first, we analyzed the sources of genetic variation at different spatial scales using GenAlEx version 6.501 [26]. The relationships among geographic subpopulations were investigated based on clone-corrected data using hierarchical analysis of molecular variance (AMOVA) at different scales, using 999 permutations. The PhiPT values, representing a 0 to 1 scaled estimator of population differentiation [32], were calculated with clone-corrected data for all pairwise comparisons for subpopulations from different provinces using 999 permutations. Principal Component Analysis (PCA) was also performed to highlight the genetic similarities and differences among the provincial subpopulations. In the second analysis, we assessed the relationships among the MLGs in the subpopulations from Fujian, Guizhou, Jiangxi, Zhejiang, and Hunan provinces, using a minimum spanning network approach to analyze the non-clone-corrected data based on Bruvo’s distance [33], using the R package *poppr*.

In the third approach, we used the program STRUCTURE v. 2.3.4 [34] to infer the number of genetic clusters in the total sample, as well as to estimate the extent to which genetic admixture might have occurred among the subpopulations from the five provinces. The STRUCTURE analyses used the admixture model, with 10 replicated runs of K = 1 to 10 after an initial burn-in of 100,000 generations followed by a run length of 1,000,000 generations. The optimal number of genetic clusters (K) was identified by following the method described in [35], using *StructureHarvester* (http://taylor0.biology.ucla.edu/structureHarvester, accessed on 1 June 2021).

At the two smaller spatial scales, we also used GenAlEx to identify the potential influences of two host species (*C. reticulata* vs. *C. sinensis* among geographically closely located orchards within Luxi county in Hunan) and two host organs (leaves vs. fruits of the same five trees within one orchard) on the observed genetic variations, respectively.

### 2.8. Mating Type Identification

The sequences of *MAT1-1-1* and *MAT1-2-1* in *D. citri* were identified by BLASTP searches against the *D. citri* genomes of strains ZJUD14 (GCA_014872985.1) and ZJUD2 (GCA_014872965.1), which contained *MAT1-1-1* and *MAT1-2-1*, respectively [22]. In these searches, we used protein sequences of *Diaporthe* W-type (*MAT1-1-1*: AB199324, *MAT1-2-1*: AB199325) and *Diaporthe* G-type (*MAT1-1-1*: AB199324, *MAT1-2-1*: AB199325) [21] as query sequences and designed the *D. citri*-specific *MAT1-1-1* primer pair DcM1F/DcM1R (5′-ATGTGGCACAAAGAAATCC-3′/5′-TCACTGGAGGTCCCAATTG-3′) and *MAT1-2-1* primer pair DcM2F/DcM2R (5′-ATGTGGCACAAAGAAATCC-3′/ 5′-TCACTGGAGGTCCCAATTG-3′), respectively, using the Primer Premier 5 software [24,36]. These primers were used to amplify the mating type loci of all 339 isolates of *D. citri* in this study. Each PCR reaction had a final volume of 25 µL, containing 1 µL of template genomic DNA (~20 ng), 1 µL forward primer, 1 µL reverse primer, 12.5 µL of× Taq Plus Master Mix (Vazyme Biotechnology Co. Hangzhou), and 9.5 µL sterile water. PCR cycling was performed as follows: 94 °C for 5 min, 35 cycles of 94 °C for 30 s, 55 °C (*MAT1-2-1*) or 56 °C (*MAT1-1-1*) for 30 s, 72 °C for 1 min, with a final extension of 7 min at 72 °C. After the amplification products of *MAT1-1-1* and *MAT1-2-1* were mixed, 4 μL of the mixture was separated on 1% agarose gel for electrophoresis (130 V, 30 min) and visualized under UV light after ethidium bromide staining.

### 2.9. Mating Type Ratios

The ratios of the *MAT1-1-1* and *MAT1-2-1* mating types among the isolates at each subpopulation and in the total population were calculated based on both the non-clone-corrected and clone-corrected datasets. A χ^2^ test with a critical value of *p* < 0.05 was performed to determine whether the mating type frequencies deviated significantly from a 1:1 ratio [37]. The null hypothesis was that the ratio of the two mating types was 1:1 (in equilibrium). Due to issues with using a χ^2^ test when analyzing small sample sizes, an exact binomial goodness-of-fit test (two-tailed) was also performed to assess whether the observed mating type ratios for analyzed populations deviated from 1:1. The χ^2^ test and the exact binomial test (two-tailed) were performed using IBM SPSS Statistics 20 (International Business Machines Corporation, Arkmonk, NY, USA). Occurrence of the two mating types in equilibrium would be an indication of sexual reproduction and recombination.

### 2.10. Analysis of Linkage Disequilibrium and Recombination

Linkage disequilibrium was analyzed by calculating the index of association (I_A_) [38] and the standardized index of association ($\bar{r}$_d_) [39] after 999 permutations in package *poppr* [31]. The null hypothesis in this test was that frequent recombination occurred and would be seen as random associations among alleles among the 14 different loci. Significant deviations from random associations would result in rejection of the null hypothesis and support the alternative hypothesis of linkage, indicating infrequent or no recombination in nature. In addition, we also tested whether the null hypothesis of no recombination would be supported. Here, the proportion of phylogenetically compatible pairs of loci (Prp) was calculated using Multilocus 1.3b with 1000 permutations. Two loci are phylogenetically compatible (Prp = 1) if they arise from the same phylogenetic position in the absence of homoplasy or recombination. Under the assumption that parallels, reversals, or convergences are rare, phylogenetic incompatibility (Prp < 1) provides the evidence of sexual recombination and exchange of genetic material between genomes [40,41]. The hypothesis of random mating would be rejected if fewer incompatible pairs of loci were observed than expected in a randomized recombining population (*p* < 0.05) [39].

## 3. Results

### 3.1. SSR Marker Development and Screening

The fourteen primer pairs we screened showed polymorphisms in the subset of 14 isolates (data not shown). The BLAST analyses of the 14 SSRs using the assembled *D. citri* genome showed that these 14 SSR loci were all located on different contigs and not closely linked. The details about these 14 loci are presented in Table 2.

### 3.2. Diversity Characteristics of the 14 SSR Markers in the D. citri Population

**Figure 1:**
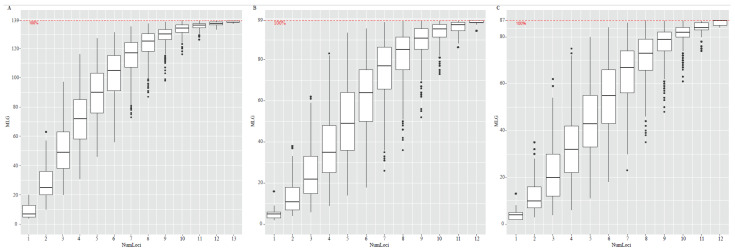
Genotype rarefaction curve for 14 SSR loci used to identify multilocus genotypes of *Diaporthe citri* populations from provincial (**A**), orchard (**B**), and tree (**C**) scales.

At the largest spatial scale that includes the five provincial populations, all 14 loci were polymorphic, and the number of alleles per locus among the 142 isolates ranged from 4 to 20, Nei’s gene diversity ranged from 0.40 to 0.89, Simpson index ranged from 0.39 to 0.89, and Evenness ranged from 0.42 to 0.86. At the next spatial scale among the four orchards used for analyzing potential local population structuring and host tree effects on pathogen gene diversity, 13 of the 14 loci were polymorphic, while the DC1 locus was monomorphic among the 102 isolates. The number of amplified alleles, Simpson index, Nei’s gene diversity, and Evenness per locus among the 102 isolates ranged from 2 to 15, 0.04 to 0.78, 0.04 to 0.79, and 0.35 to 0.88 in the orchard population, respectively (Table 3). Similarly, 13 of the 14 loci were polymorphic among the 95 isolates from the five trees in the same orchard, with locus DC1 being monomorphic. The number of amplified alleles, Simpson index, Nei’s gene diversity, and Evenness per locus ranged from 2 to 13, 0.11 to 0.80, 0.11 to 0.81, and 0.39 to 0.89 in the tree population, respectively (Table 3). The genotype rarefaction curve showed increases in the number of genotypes as the number of the loci increased at all three examined spatial scales and reached a plateau at 13 loci at the provincial level, and at 12 loci at the orchard and tree levels (Figure 1). Together, these results indicate that the 14 SSR markers were sufficient to discriminate among strains at the three spatial scales.

The above results on gene diversities described the variations among the 14 loci at different spatial scales. When all loci are considered, individual populations at all three spatial scales also showed high diversities. For example, the numbers of different and effective alleles per locus ranged from 2.957 (tree level) to 4.104 (provincial level) and 1.920 (orchard level) to 2.466 (provincial level); the Shannon’s information indices were 0.908, 0.684, and 0.681; and the unbiased Nei’s diversities were 0.472, 0.348, and 0.363 for populations at provincial, orchard, and tree scales, respectively. The values of unbiased allelic diversities were overall similar to Nei’s diversity (Table 4).

Among the five provincial subpopulations, that from Fujian Province showed the highest gene diversity, with Nei’s gene diversity and Shannon’s information index being 0.638 and 1.252, respectively, followed by the Zhejiang subpopulation with Nei’s gene diversity and Shannon’s information index at 0.569 and 1.132, respectively. The levels of gene diversity of the Hunan, Jiangxi, and Guizhou subpopulations were similar, with Nei’s gene diversity and Shannon’s information index ranging from 0.403 to 0.424 and 0.839 to 0.874, respectively (Table 4).

**Figure 2:**
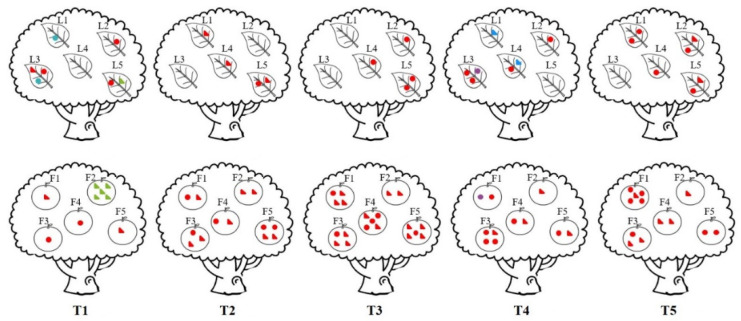
Distribution of two mating types within and among trees. (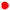) represents *MAT1-1-1* isolates, (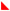) represents *MAT1-2-1* isolates. The isolates in red color have unique MLGs. Isolates in other colors have shared MLGs with isolates of the same colors. T1~T5 indicate tree1~tree5, L1~L5 indicate leaf 1~leaf 5, F1~F5 indicate fruit 1~fruit 5.

Similar to gene diversity analyses, results from the genotype diversity analysis also revealed high genotypic diversities within all examined populations, with 139 (98%), 99 (97%), and 87 (92%) MLGs identified among 142, 102, and 95 fungal isolates analyzed from the provincial, orchard, and tree levels, respectively (Table 4). At the provincial level, three of the 139 MLGs were shared, with each shared MLG found between two isolates from different trees in the same orchard. One shared MLG each was found in Guizhou, Hunan, and Zhejiang provinces (Table 4). Similarly, at the orchard level, three of the 99 MLGs were shared, with each shared MLG found between two isolates from different trees in the same orchard. Two of the shared MLGs were in orchard 1 and the third shared MLG was found in orchard 2 (O2 in Table 4). Interestingly, among the five trees within the same orchard, there was no shared MLG in our sample—all the shared MLGs were found among isolates either from different leaves/fruits on the same tree or from different melanose spots on the same leaf and fruit (Figure 2).

### 3.3. Genetic Structure among Populations of D. citri

**Figure 3:**
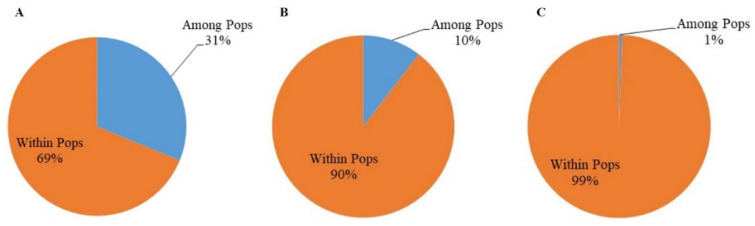
Analysis of molecular variance for *Diaporthe citri* subpopulations at the provincial (**A**), orchard (**B**), and tree (**C**) levels.

**Figure 4:**
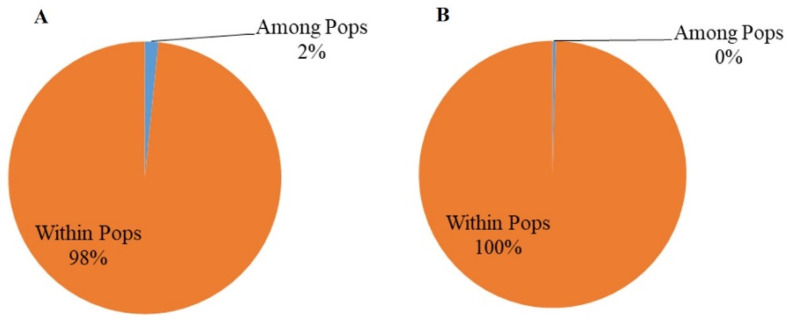
Analysis of molecular variance for *Diaporthe citri* populations between host tree species-based populations at orchard scale (**A**) and between organ type-based populations at the tree scale (**B**).

AMOVA analysis showed that 31% of the genetic variation was distributed among the subpopulations from the five provinces, and 69% was distributed within the subpopulations. At the orchard level, 10% of the total genetic variations were distributed among the four orchards, while 90% were found within the orchards, with less than 2% coming from between the two citrus species (*p* > 0.05), consistent with limited host tree specialization in *D. citri* genotypes. At the tree level within the orchard in Luxi, 1% of the total genetic variations were found among the subpopulations from the five trees, while 99% were found within the individual tree subpopulations (Figure 3). Furthermore, there was no evidence of genetic differentiation between *D. citri* populations from the two different organ types (leaf vs. fruit) (Figure 4).

**Figure 5:**
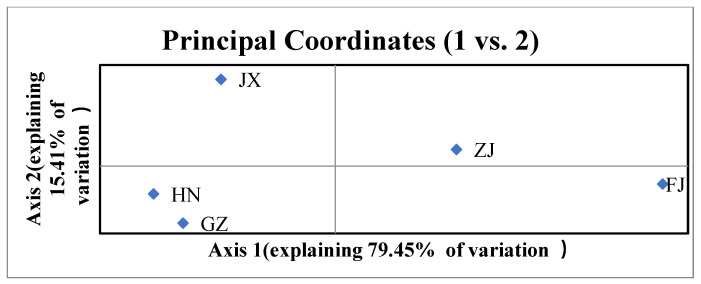
Principal component analysis for subpopulations from five provinces. FJ, GZ, JX, ZJ, and HN represent Fujian, Guizhou, Jiangxi, Zhejiang, and Hunan provinces, respectively. The data are clone-corrected.

**Figure 6:**
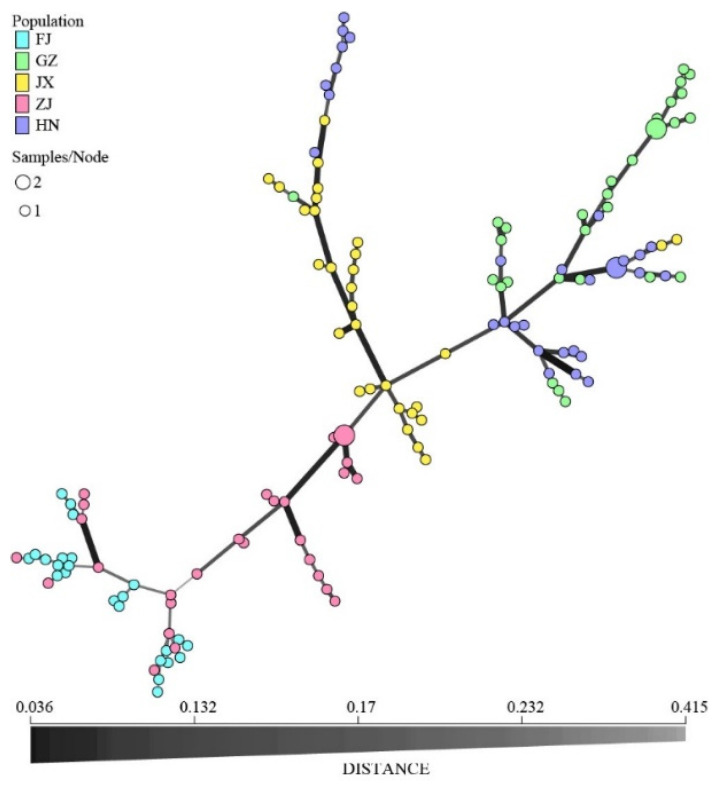
Minimum spanning network for subpopulations from five provinces. FJ, GZ, JX, ZJ, and HN represent Fujian, Guizhou, Jiangxi, Zhejiang, and Hunan provinces, respectively. The different colored circles represent the individuals of different provincial subpopulations, and the circle size represents the number of isolates with the same MLG. The data are clone-corrected.

Pairwise PhiPT values showed significant genetic differentiation between all provincial subpopulations (*p* = 0.001), with the PhiPT values ranging from 0.104 (HN vs. GZ) to 0.421 (FJ vs. JX) (Table 5). PCA analysis revealed that the subpopulations from five provinces were divided into two genetic groups. One group included subpopulations from Hunan, Guizhou, and Jiangxi, while the other group included subpopulations from Fujian and Zhejiang (Figure 5). The minimum spanning network revealed that the MLGs from Fujian and Zhejiang are distinct from the Hunan, Guizhou, and Jiangxi subpopulations (Figure 6).

**Figure 7:**
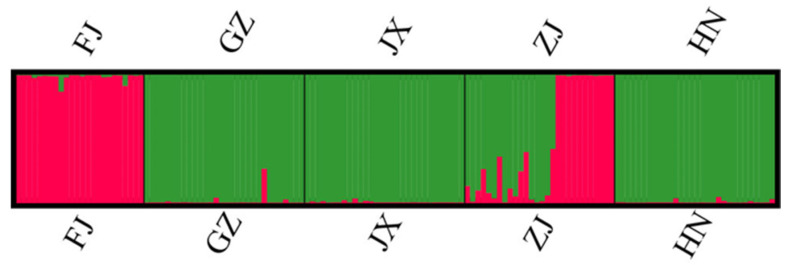
Structure results of the five provincial subpopulations. Different colors represent different genetic clusters (K = 2) with each vertical line representing an isolate. Data were clone-corrected.

The STRUCTURE analysis also identified two genetic clusters (K = 2). However, isolates in the two genetic clusters showed different frequency distributions among the five provincial subpopulations. Isolates of one genetic cluster were distributed mostly in the Fujian subpopulations, and the other genetic cluster was mainly distributed in subpopulations from Hunan, Jiangxi, and Guizhou provinces. The Zhejiang subpopulation had similar representations of both genetic clusters (Figure 7). The results of the STRUCTURE analysis are consistent with the results of the pairwise PhiPT comparisons, the PCA, and minimum spanning network (MSN), which indicated low-level gene flow among five provinces’ subpopulations. Interestingly, isolates with genetic elements of both clusters were found in all five provinces, consistent with the presence of hybridization among these geographic populations.

Mantel analysis showed significant correlation between genetic and geographic distances (r = 0.7228, *p* = 0.001), consistent with geographic separation playing a significant role in the genetic differentiation among these five provincial populations.

### 3.4. Mating Type Identification and Ratios

A positive PCR amplification was obtained for all isolates with either primer pair DcM1F/DcM1R, which is specific to isolates of mating type *MAT1-1*, or primer pair DcM2F/DcM2R, which is specific to isolates of mating type *MAT1-2*. In populations at all three spatial scales of analyses, the mating type frequencies did not deviate significantly from a 1:1 ratio based on the χ^2^ test (non-clone-corrected data, 0.166 ≤ *p* ≤ 0.918; clone-corrected data, 0.132 ≤ *p* ≤ 0.932) and the exact binomial analysis (non-clone-corrected data, 0.198 ≤ *p* ≤ 1; clone-corrected data, 0.159 ≤ *p* ≤ 1) (Table 6). Indeed, within each subpopulation from a province, orchard, and tree, the mating types were also in similar frequencies (Table 6). Furthermore, the two mating types were found to co-exist on 13 of the 19 (68%) fruits and 6 of the 9 (67%) leaves from which at least two isolates were obtained for analyses (Figure 2).

### 3.5. Linkage Disequilibrium and Recombination

For the original non-clone-corrected data, most (16/21) of the subpopulations did not reject the null hypothesis of linkage equilibrium (*p* > 0.05), except for five subpopulations: GZ (*p* = 0.001), ZJ (*p* = 0.001), O2 (*p* = 0.012), T1 (*p* = 0.001), and T5 (*p* = 0.015). Similarly, Prp values were consistent with phylogenetic incompatibility in most of the subpopulations (*p* > 0.05), except for FJ (*p* = 0.020), GZ (*p* = 0.025), ZJ (*p* = 0.001), T1 (*p* = 0.010), T4 (*p* = 0.001), and T5 (*p* = 0.043). In the clone-corrected data, the T1 subpopulation was in linkage equilibrium (*p* = 0.126), with a Prp value of 0.807. For both the non-clone-corrected and clone-corrected data, phylogenetic incompatibility was found in all subpopulations (Table 7). These results provided strong evidence that *D. citri* underwent sexual reproduction in these natural populations. However, the weak but statistically significant linkage disequilibrium observed in some subpopulations also suggests evidence of genetic linkage and clonal reproduction.

### 3.6. Genetic Diversity of the Two MAT Idiomorph Populations

**Figure 8:**
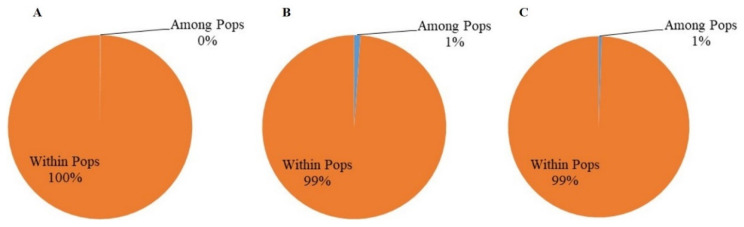
Analysis of molecular variance between the *MAT1-1* and *MAT1-2* subpopulations of *Diaporthe citri* at three spatial scales of provincial (**A**), orchard (**B**), and tree levels (**C**).

**Figure 9:**
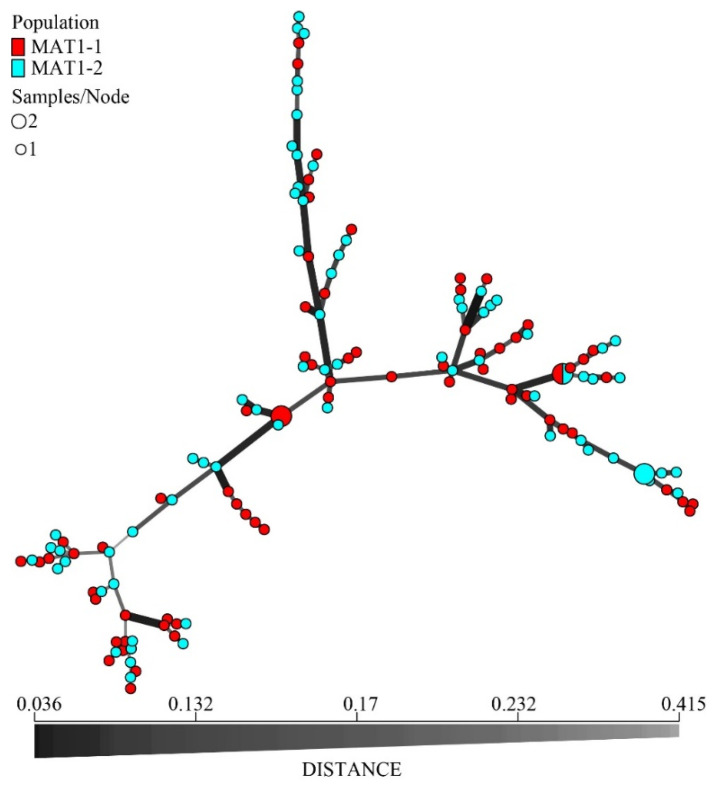
Minimum spanning network based on pairwise Bruvo’s genetic distances for isolates belonging to the two mating types at the provincial level. Node size indicates the number of strains of the specific MLG and the branch lengths indicate genetic distance between isolates.

Allele diversity analyses, including the number of different alleles, the number of effective alleles, Shannon’s information indices, Nei’s gene diversities, and unbiased allelic diversities between the *MAT1-1* and *MAT1-2* subpopulations, showed high and similar levels of genetic diversity at all three spatial levels (Table 8). For both the non-clone-corrected and clone-corrected dataset, AMOVA showed that less than 1% of the genetic variation could be explained by differences between the *MAT1-1* and *MAT1-2* subpopulations at all three spatial scales (Figure 8). Furthermore, the minimum spanning network revealed that the MLGs of two mating types were intermixed at all three of the studied scales (Figure 9). Together, these results are consistent with frequent mating and recombination in natural populations of *D. citri* in citrus orchards.

## 4. Discussion

Melanose caused by *D. citri* is an important disease of citrus in southern China and has significantly influenced the marketability of citrus fruits [3]. In this study, we analyzed the patterns of genetic variation of *D. citri* in orchards of *C. reticulata* and *C. sinensis* to understand the mode of reproduction of *D. citri* in nature and to reveal the relationships among populations of *D. citri* from different trees, orchards, and provinces in southern China. Consistent with what we hypothesized, geographic separation was found to have played a significant role in structuring the genetic diversity of *D. citri* in southern China. In addition, as was hypothesized, limited differences were found between host tree species-based subpopulations and between plant organ-based subpopulations. However, different from what we expected, both linkage disequilibrium analysis and phylogenetic incompatibility tests revealed abundant evidence for recombination within individual subpopulations. Furthermore, the ratios of the two mating types *MAT1-1-1* and *MAT1-2-1* in both the total sample and in most subpopulations of *D. citri* analyzed here did not deviate significantly from 1:1. Together, the genetic signatures of the *D. citri* populations in southern China are consistent with this pathogen being highly endemic in these regions [19,41].

Our analyses revealed that geographical isolation played a significant role in population differentiations among the five provincial populations of *D. citri.* The influences of geography on population structure have been reported in many other plant fungal pathogens such as the wheat pathogen *Mycosphaerella graminicola* [42], the chili pepper anthracnose pathogen *Colletotrichum truncatum* [43], the grapevine dieback pathogen *Diaporthe* spp. [15], the yam wilt pathogen *Fusarium oxysporum* species complex [44], and the tea-oil tree anthracnose pathogen *Colletotrichum fructicola* [45]. Indeed, in southern China, a diversity of genetic relationships among regional populations has been observed for plant fungal pathogens, e.g., [43,44,45]. For example, multilocus sequence typing revealed significant genetic differentiations among regional populations of *C. fructicola* from tea-oil tree plantations in Hunan and Jiangxi provinces [45]. However, long-distance dispersals were also found, likely related to tea-oil tree seedling trade among regions by humans [45]. Long-distance sharing of multilocus genotypes has also been reported for other fungi in southern China, including the human fungal pathogens *Cryptococcus neoformans* [46] and *Candida tropicalis* [47], likely due to anthropogenic influences on these fungal pathogens. However, different from most previous studies, no shared multilocus genotype was found in this study among isolates of *D. citri* from the five provinces in southern China. The result suggested one of two possibilities. In the first, long-distance dispersal of asexual spores of *D. citri* was uncommon. In the second, long-distance dispersal was common but after dispersal, sexual mating and recombination were frequent enough to break the allelic associations in these asexual propagules to generate new genotypes. Based on the observed allelic distributions among geographic populations at most loci, it seemed that the second possibility was more likely than the first. However, with increased trade and human travel among these provinces, we will likely observe long-distance dispersals of *D. citri* among geographic populations.

We found that the two mating types (*MAT1-1-1* and *MAT1-2-1*) were distributed at similar frequencies in most subpopulations of *D. citri* at all three spatial scales. In combination with results from linkage equilibrium and Prp analyses, this result is consistent with the frequent mating and recombination of *D. citri* in nature. Similar results have been reported in several plant fungal pathogens such as *Phyllosticta citricarpa* [48,49], *Zymoseptoria tritici* [50], *Dothistroma septosporum* [51], and *C. truncatum* [43]. High genetic diversity is typical of sexually reproducing species [18,20,41]. Carstens et al. [49] studied the global genetic diversity of *P. citricarpa,* the causal agent of citrus black spot, and found that the populations from China and Australia were sexually reproducing populations with very high genetic diversity. In contrast, the population from United States was consistent with asexual reproduction, with only one multilocus genotype (MLG), and all strains had the same mating type, *MAT1-2-1*. Our research showed that *D. citri* populations have high levels of genetic diversity at all three studied spatial scales in China, likely due to frequent sexual recombination in orchards. Similar results have been reported another citrus fungal pathogen *P. citriasiana* [52].

Interestingly, we observed very few shared MLGs among the 339 isolates. Previous studies have suggested that the large number of conidia of *D. citri* produced on dead twigs are likely the infectious propagules that are transmitted to healthy fruits and leaves through rain or irrigation water to cause new infections [53]. If this hypothesis were true, we should observe frequent MLG sharing throughout the same tree and the same orchard. However, our study found very few shared MLGs of *D. citri,* even in a very small spatial area. Therefore, we speculate that although *D. citri* can produce a large number of conidia in the orchard, the infection rate on citrus by conidia is likely low, with most of the lesions not caused by conidia but likely by sexual spores (ascospores).

A previous study suggested that *D. citri* likely has multiple modes of nutrient acquisition, as a biotroph that can cause diseases, a saprophyte that grows on a variety of dead branches and twigs, and as endophyte living under the epidermis in the intercellular space of trees [2,7,8]. As most citrus trees are perennial plants, the *D. citri* populations could have existed in these orchards for an extended time and represented populations in relative equilibrium. In such equilibrium populations, sexual reproduction and local adaptation are likely common for populations of *D. citri*, potentially making it difficult for immigrants to establish in the endemic niches. However, the observation of significant genetic differentiations among the five provincial populations does not mean that there was no migration. As shown in the minimum spanning tree, closely related strains were found from several geographically distinct areas. In addition, STRUCTURE analyses identified evidence for genetic admixture between the two highly differentiated genetic clusters in most geographic populations (but most prevalent in Zhejiang Province). Together, these results suggest that the *D. citri* populations are likely changing. With increasing human influences, both directly through the exchange of citrus seedlings and indirectly by impacting climate change, the changes in *D. citri* populations will likely accelerate.

Though the sample size was relatively large, our study only analyzed samples of *D. citri* from two *Citrus* tree species in 21 orchards in five provinces in southern China. Many issues about the ecology, evolution, and population genetics remain unanswered. For example, at present, the exact host range of *D. citri* outside citrus trees remains undefined. Further sampling and analyses of *D. citri* populations from host plants outside of *Citrus* species, from other *Citrus* species (i.e., species other than *C. reticulata* and *C. sinensis* that we analyzed here), and from decaying twigs on the ground over time are needed in order to provide a more robust understanding on the origins and epidemiology of the *D. citri* populations causing citrus melanose.

## Figures and Tables

**Table 1:** Information about population samples of *Diaporthe citri* collected from citrus orchards in southern and southeastern China in 2019 for this study.

Populations	Source of Location	Longitude	Latitude	Host	Designation of Population	Number of Isolates
Province	Fujian				FJ	24
	Shunchang A	117.94° E	27.12° N	*Citrus reticulata*		3
	Shunchang B	117.90° E	27.12° N			1
	Songxi A	117.89° E	27.03° N			1
	Songxi B	116.41° E	27.05° N			19
	Guizhou				GZ	30
	Congjiang A	108.88° E	25.67° N	*C. reticulata*		10
	Congjiang B	108.82° E	25.71° N			9
	Congjiang C	108.84° E	25.71° N			11
	Jiangxi				JX	30
	Jingan A	115.36° E	28.85° N	*C. reticulata*		8
	Jingan B	115.30° E	28.85° N			10
	Jingan C	115.39° E	28.81° N			7
	Jingan D	115.40° E	28.82° N			5
	Zhejiang				ZJ	28
	Kecheng A	118.82° E	28.97° N	*C. reticulata*		6
	Kecheng B	118.87° E	29.02° N			1
	Kecheng C	118.86° E	29.05° N			1
	Qujiang	118.89° E	29.00° N			9
	Huangyan	121.16° E	28.63° N			11
	Hunan				HN	30
	Luxi A	110.04° E	28.24° N	*C. reticulata*		14
	Luxi B	109.97° E	28.26 N			16
Subtotal						142
Orchard	Hunan					
	Luxi A	110.05° E	28.24° N	*C. reticulata*	O1	25
	Luxi B	109.97° E	28.26° N	*C. reticulata*	O2	24
	Luxi C	110.04° E	28.24° N	*C. sinensis*	O3	28
	Luxi D	110.10° E	28.25° N	*C. sinensis*	O4	25
Subtotal						102
Tree	Luxi E	109.97° E	28.23° N	*C. reticulata*	T1	16
	Luxi E	109.97° E	28.23° N	*C. reticulata*	T2	17
	Luxi E	109.97° E	28.23° N	*C. reticulata*	T3	24
	Luxi E	109.97° E	28.23° N	*C. reticulata*	T4	18
	Luxi E	109.97° E	28.23° N	*C. reticulata*	T5	20
Subtotal						95

**Table 2:** Information of primer pairs and product size range for the 14 SSR loci analyzed in this study.

Locus	Repeat Motif	Primer Sequence (5′ to 3′)	Product Size	OAT ^1^	Labeling Dye
DC8	(TG)_16_	F: GTGCTTCTCTTGTTGTTTGTT	128–250	55 °C	FAM
		R: AACTTTCCTTTCCCTTCTTCT			
DC13	(GGT)_9_	F: GGTGGAGATGAACCCTTC	124–151	53 °C	FAM
		R: CGCCTGGTACGTTACATT			
DC2	(AG)_9_	F: TGAATTGGGAGAAAGAGAGAC	121–141	56 °C	FAM
		R: TGGTAACAGACCAAGATGATT			
DC31	(CA)_20_	F: GACTATCGCACTTACAACTGG	157–197	55 °C	FAM
		R: ACTTCCAAAGGGTATTTGTGT			
DC41	(CTGC)_11_	F: AAACTGCTGCAGAACATCTC	144–196	56 °C	TAMRA
		R: GATCTGGGTCAGGATCGTA			
DC10	(TG)_8_	F: GATGGAGAACACAGAGATGAA	214–264	55 °C	TAMRA
		R: ATATCAACATTCTGACCACCA			
DC39	(CA)_29_	F: GTCTGTCTCCTTGTGTCTCTC	100–156	55 °C	TAMRA
		R: AAACGTTCCCCTTCTCTAAC			
DC54	(AC)_33_	F: AGTCCGTCCAATGTCTGTC	99–193	55 °C	TAMRA
		R: TGCATATGTTGTGCTTTGTT			
DC43	(CT)_33_	F: CTCTCTCACACACACACACAC	130–238	53 °C	TAMRA
		R: TAGCTGTAGATGAGTCCCAGA			
DC14	(CA)_8_	F: CTCCCTCTTCCCAGACAC	156–214	55 °C	HEX
		R: AGAGGAGGTTGTATTTTGTGTT			
DC27	(CACT)_12_	F: AAATCACCCCACACTCAAC	110–138	55 °C	HEX
		R: AGACATGGAGCAACACAAGT			
DC4	(CAG)_10_	F: ATCACACCTTTCATCTCATCA	136–157	55 °C	HEX
		R: CGAGAGTTTCTCTCCTTGG			
DC1	(AG)_11_	F: CTCCAGCATGACATAGTTAGG	156–170	53 °C	HEX
		R: CTGGTCTGATCTGTAAGTGGA			
DC55	(AC)_9_	F: ATTTCCTCTCAAGCACAATG	154–282	55 °C	HEX
		R: ATGGTCCAGTCACAAACATAG			

^1^ OAT, optimal annealing temperature.

**Table 3:** Polymorphism of 14 SSR loci at the provincial, orchard, and tree populations of *D. citri* ^1^.

Locus	Province				Orchard				Tree			
	Na	λ	Hexp	E	Na	λ	Hexp	E	Na	λ	Hexp	E
DC1	6	0.67	0.68	0.69	6	0.74	0.75	0.85	5	0.64	0.65	0.84
DC2	4	0.45	0.45	0.65	4	0.46	0.46	0.68	2	0.19	0.19	0.58
DC4	4	0.57	0.57	0.74	1	0.00	0.00	NA	1	0.00	0.00	NA
DC8	20	0.84	0.84	0.62	15	0.78	0.79	0.64	13	0.79	0.80	0.65
DC10	13	0.84	0.85	0.78	9	0.64	0.64	0.57	8	0.80	0.81	0.83
DC13	5	0.39	0.40	0.55	3	0.10	0.10	0.41	2	0.11	0.11	0.49
DC14	13	0.89	0.89	0.86	8	0.70	0.71	0.66	8	0.61	0.61	0.54
DC27	5	0.65	0.66	0.77	3	0.06	0.06	0.37	2	0.11	0.11	0.49
DC31	10	0.53	0.54	0.45	2	0.04	0.04	0.40	4	0.11	0.11	0.39
DC39	12	0.79	0.79	0.71	5	0.74	0.74	0.88	5	0.72	0.73	0.89
DC41	7	0.68	0.68	0.73	4	0.32	0.32	0.58	5	0.39	0.39	0.51
DC43	14	0.75	0.75	0.61	5	0.62	0.62	0.79	4	0.57	0.58	0.84
DC54	11	0.50	0.51	0.42	5	0.10	0.10	0.35	3	0.17	0.17	0.50
DC55	5	0.58	0.58	0.71	2	0.27	0.27	0.67	2	0.22	0.22	0.61
Mean	9	0.65	0.66	0.66	5	0.40	0.40	0.60	5	0.39	0.39	0.63

^1^ Calculated based on clone-corrected data. Na, Number of alleles; λ, Simpson index; Hexp, Nei’s gene diversity; E, Evenness, the distribution of genotype abundance.

**Table 4:** Genetic diversity of *Diaporthe citri* subpopulations at three different spatial scales in China ^1^.

Scale	Subpopulations	N	MLGs	eMLGs	Ne	I	h	uH
Provincial	FJ	24	24	24.00	3.254	1.252	0.638	0.665
	GZ	30	29	23.37	2.289	0.809	0.420	0.435
	JX	30	30	24.00	1.840	0.604	0.350	0.362
	ZJ	28	27	23.27	2.780	1.132	0.569	0.591
	HN	30	29	23.37	2.165	0.745	0.382	0.395
	Total	142	139	23.92	2.466	0.908	0.472	0.490
Orchard	O1	25	23	22.16	1.988	0.729	0.378	0.395
	O2	24	23	23.00	1.923	0.677	0.348	0.363
	O3	28	28	24.00	2.061	0.736	0.374	0.388
	O4	25	25	24.00	1.768	0.610	0.314	0.327
	Total	102	99	23.84	1.920	0.684	0.348	0.360
Tree	T1	16	10	10.00	1.907	0.585	0.314	0.349
	T2	17	17	16.00	2.099	0.704	0.374	0.397
	T3	24	24	16.00	2.120	0.711	0.375	0.391
	T4	18	16	14.43	2.273	0.771	0.409	0.436
	T5	20	20	16.00	1.935	0.637	0.346	0.364
	Total	95	87	15.58	2.067	0.681	0.363	0.388

^1^ Data on Ne, I, h, and uh in this table were based on clone-corrected samples. MLGs, multilocus genotypes; eMLGs, expected multilocus genotypes; N, Number of isolates; Ne, Number of effective alleles; I, Shannon’s information index; H, Nei’s gene diversity (Nei, 1973) across all loci; uH, Nei’s unbiased genotypic diversity. MLG and eMLG were calculated in R v. 3.6.3 using the analysis package *poppr*, and Ne, I, H, and uH were calculated in GenAlEx version 6.502.

**Table 5:** PhiPT values among five provincial subpopulations of *Diaporthe citri* in southern China ^1^.

Subpopulations	FJ	GZ	JX	ZJ	HN
FJ	0.000	0.001	0.001	0.001	0.001
GZ	0.387	0.000	0.001	0.001	0.001
JX	0.421	0.323	0.000	0.001	0.001
ZJ	0.144	0.299	0.298	0.000	0.001
HN	0.419	0.104	0.292	0.325	0.000

^1^ Data were clone-corrected. The values above the diagonal line indicate the *p* values. The values below the diagonal line are the PhiPT values.

**Table 6:** Ratio of the two mating types within subpopulations at three different spatial scales.

Scale	Subpopulation	Raw Data				Clone-Corrected Data			
		*MAT1-1*	*MAT1-2*	χ^2^ (*p*) ^a^	*p* ^b^	*MAT1-1*	*MAT1-2*	χ^2^ (*p*)	*p*
Province	FJ	11	13	0.167 (0.683)	0.839	11	13	0.167 (0.683)	0.839
	GZ	15	15	0.000 (1)	1	15	14	0.034(0.853)	1
	JX	13	17	0.533 (0.465)	0.585	13	17	0.533 (0.465)	0.585
	ZJ	17	11	1.286 (0.257)	0.345	16	11	0.926(0.336)	0.442
	HN	14	16	0.133 (0.715)	0.856	14	15	0.034(0.853)	1
	Total	70	72	0.028 (0.867)	0.933	69	70	0.007(0.932)	1
Orchard	O1	9	16	1.960 (0.162)	0.23	8	15	2.130(0.144)	0.21
	O2	14	10	0.667 (0.414)	0.541	13	10	0.391(0.532)	0.678
	O3	12	16	0.571(0.450)	0.572	12	16	0.571(0.450)	0.572
	O4	9	16	1.960(0.162)	0.23	9	16	1.960(0.162)	0.23
	Total	44	58	1.992(0.166)	0.198	42	57	2.273(0.132)	0.159
Tree	T1	7	9	0.250(0.617)	1	6	4	0.40(0.527)	0.754
	T2	6	11	1.471 (0.225)	0.332	6	11	1.471 (0.225)	0.332
	T3	10	14	0.667(0.414)	0.541	10	14	0.667(0.414)	0.541
	T4	12	6	2.000 (0.157)	0.238	11	5	2.25(0.134)	0.21
	T5	12	8	0.8 (0.371)	0.503	12	8	0.8 (0.371)	0.503
	Total	47	48	0.011 (0.918)	1	45	42	0.103(0.748)	0.83

^a^ The *p* values in parentheses indicate if the ratio was significantly different to 1:1 (significant if *p* ≤ 0.05), calculated in IBM SPSS Statistics 20 (International Business Machines Corporation, Armonk, NY, USA). ^b^ Probability from a two-tailed exact binomial analysis to test whether MAT frequencies deviate significantly from a 1:1 ratio, calculated in IBM SPSS Statistics 20 (International Business Machines Corporation, Armonk, NY, USA).

**Table 7:** Linkage disequilibrium and recombination analyses for *Diaporthe citri* populations from different scales in China ^1^.

Scale	Subpopulation	Raw Data						Clone-Corrected Data					
		N	I_A_	$\bar{r}$ _d_	*p* ^2^	Prp	*p* ^3^	N	I_A_	$\bar{r}$ _d_	*p*	Prp	*p*
Province	FJ	24	0.098	0.008	0.171	0.044	0.020	24	0.098	0.008	0.144	0.044	0.020
	GZ	30	0.531	0.050	0.001	0.484	0.025	29	0.484	0.045	0.003	0.484	0.025
	JX	30	0.024	0.002	0.383	0.527	0.735	30	0.024	0.002	0.371	0.527	0.735
	ZJ	28	2.322	0.195	0.001	0.396	<0.001	27	2.201	0.185	0.001	0.396	<0.001
	HN	30	0.123	0.013	0.125	0.626	0.544	29	0.074	0.008	0.238	0.626	0.514
	Total	142	2.115	0.166	0.001	0.000	1.000	139	2.091	0.164	0.001	0.000	1.000
Orchard	O1	25	0.070	−0.007	0.682	0.648	0.510	23	−0.142	−0.013	0.830	0.648	0.769
	O2	24	0.332	0.034	0.012	0.725	0.840	23	0.259	0.027	0.043	0.725	0.822
	O3	28	0.104	0.010	0.219	0.615	0.709	28	0.104	0.010	0.219	0.615	0.709
	O4	25	0.167	0.018	0.151	0.736	0.415	25	0.167	0.018	0.151	0.736	0.415
	Total	102	0.120	0.011	0.030	0.330	0.794	99	0.113	0.010	0.041	0.330	0.766
Tree	T1	16	1.550	0.198	0.001	0.923	0.010	10	0.319	0.041	0.126	0.923	0.807
	T2	17	0.072	0.007	0.293	0.670	0.280	17	0.072	0.007	0.307	0.670	0.280
	T3	24	0.137	0.013	0.156	0.593	0.595	24	0.137	0.013	0.159	0.593	0.595
	T4	18	0.230	0.020	0.096	0.670	0.001	16	−0.038	−0.003	0.565	0.670	0.028
	T5	20	0.389	0.037	0.015	0.769	0.043	20	0.389	0.037	0.019	0.769	0.043
	Total	95	0.158	0.014	0.014	0.275	0.514	87	0.105	0.009	0.074	0.275	0.572

^1^ N: number of isolates; I_A_: index of association; $\bar{r}$_d_: standard index of association; Prp: Proportion of compatible pairs of loci; ^2^ *p* values of I_A_ and $\bar{r}$_d_, calculated in package *poppr* with 999 permutations; ^3^ *p* values of Prp, calculated in Multilocus 1.3b with 1000 permutations.

**Table 8:** Allele diversity of two mating type-based subpopulations at three different spatial scales ^1^.

Scale	Pops	Subpops	N	Na	Ne	I	h	uh
Province	FJ	*MAT1-1*	11	4.143	3.031	1.142	0.600	0.660
		*MAT1-2*	13	4.286	3.077	1.187	0.628	0.680
	GZ	*MAT1-1*	15	3.214	2.231	0.768	0.408	0.437
		*MAT1-2*	14	2.786	2.081	0.666	0.370	0.398
	JX	*MAT1-1*	13	2.214	1.784	0.535	0.313	0.339
		*MAT1-2*	17	2.357	1.810	0.592	0.356	0.379
	ZJ	*MAT1-1*	16	4.357	3.033	1.153	0.597	0.637
		*MAT1-2*	11	3.357	2.198	0.842	0.451	0.496
	HN	*MAT1-1*	14	2.786	2.080	0.659	0.355	0.382
		*MAT1-2*	15	2.929	2.078	0.693	0.374	0.401
	Total	*MAT1-1*	69	7.571	3.502	1.405	0.645	0.654
		*MAT1-2*	70	8.071	3.716	1.428	0.648	0.658
Orchard	O1	*MAT1-1*	8	2.286	1.781	0.576	0.342	0.390
		*MAT1-2*	15	3.214	2.077	0.722	0.377	0.404
	O2	*MAT1-1*	13	2.714	1.889	0.576	0.298	0.323
		*MAT1-2*	10	2.643	1.872	0.649	0.370	0.411
	O3	*MAT1-1*	12	2.929	1.912	0.681	0.369	0.403
		*MAT1-2*	16	2.929	2.007	0.645	0.338	0.361
	O4	*MAT1-1*	9	2.071	1.701	0.478	0.280	0.315
		*MAT1-2*	15	2.929	1.709	0.599	0.310	0.332
	Total	*MAT1-1*	42	4.429	2.204	0.801	0.398	0.408
		*MAT1-2*	57	4.357	2.136	0.780	0.385	0.392
Tree	T1	*MAT1-1*	6	2.000	1.659	0.454	0.266	0.319
		*MAT1-2*	4	1.929	1.743	0.482	0.295	0.393
	T2	*MAT1-1*	6	2.429	2.139	0.644	0.365	0.438
		*MAT1-2*	11	2.571	1.937	0.624	0.351	0.386
	T3	*MAT1-1*	10	2.357	1.940	0.603	0.347	0.386
		*MAT1-2*	14	2.786	2.039	0.654	0.355	0.382
	T4	*MAT1-1*	11	2.929	2.276	0.756	0.410	0.451
		*MAT1-2*	5	2.143	1.970	0.568	0.337	0.421
	T5	*MAT1-1*	12	2.571	1.954	0.632	0.357	0.390
		*MAT1-2*	8	2.071	1.602	0.448	0.254	0.291
	Total	*MAT1-1*	45	4.000	2.192	0.790	0.397	0.406
		*MAT1-2*	42	3.786	2.116	0.725	0.361	0.370

^1^ Data were clone-corrected. N, Number of isolates; Na, Number of different alleles; Ne, Number of effective alleles; I, Shannon’s information index; H, Nei’s gene diversity (Nei, 1973) across all loci; uH, Nei’s unbiased genotypic diversity. Na, Ne, I, H, and uH were calculated in GenAlEx version 6.502.

## Data Availability

Not applicable.
